# Agreement between standard and self-reported assessments of physical frailty syndrome and its components in a registry of community-dwelling older adults

**DOI:** 10.1186/s12877-022-03376-x

**Published:** 2022-08-25

**Authors:** Brian Buta, Scott Zheng, Jackie Langdon, Bukola Adeosun, Karen Bandeen-Roche, Jeremy Walston, Qian-Li Xue

**Affiliations:** 1 Center On Aging and Health, Johns Hopkins University, 2024 E. Monument Street, Suite 2-700, MD 21205 Baltimore, USA; 2 Department of Medicine, Division of Geriatric Medicine and Gerontology, Johns Hopkins University School of Medicine, Baltimore, USA; 3 Duke University School of Medicine, Durham, USA; 4 Department of Biostatistics, Johns Hopkins Bloomberg School of Public Health, Baltimore, USA

**Keywords:** Frailty, Self-reported, Agreement, Phenotype, Clinical utility

## Abstract

**Background:**

The ability to identify frail older adults using a self-reported version of the physical frailty phenotype (PFP) that has been validated with the standard PFP could facilitate physical frailty detection in clinical settings.

**Methods:**

We collected data from volunteers (*N* = 182), ages 65 years and older, in an aging research registry in Baltimore, Maryland. Measurements included: standard PFP (walking speed, grip strength, weight loss, activity, exhaustion); and self-reported questions about walking and handgrip strength. We compared objectively-measured gait speed and grip strength to self-reported questions using Cohen’s Kappa and diagnostic accuracy tests. We used these measures to compare the standard PFP with self-reported versions of the PFP, focusing on a dichotomized identification of frail versus pre- or non-frail participants.

**Results:**

Self-reported slowness had fair-to-moderate agreement (Kappa(k) = 0.34–0.56) with measured slowness; self-reported and objective weakness had slight-to-borderline-fair agreement (k = 0.10–0.21). Combining three self-reported slowness questions had highest sensitivity (81%) and negative predictive value (NPV; 91%). For weakness, three questions combined had highest sensitivity (72%), while all combinations had comparable NPV. Follow-up questions on level of difficulty led to minimal changes in agreement and decreased sensitivity. Substituting subjective for objective measures in our PFP model dichotomized by frail versus non/pre-frail, we found substantial (k = 0.76–0.78) agreement between standard and self-reported PFPs. We found highest sensitivity (86.4%) and NPV (98.7%) when comparing the dichotomized standard PFP to a self-reported version combining all slowness and weakness questions. Substitutions in a three-level model (frail, vs pre-frail, vs. non-frail) resulted in fair-to-moderate agreement (k = 0.33–0.50) with the standard PFP.

**Conclusions:**

Our results show potential utility as well as challenges of using certain self-reported questions in a modified frailty phenotype. A self-reported PFP with high agreement to the standard phenotype could be a valuable frailty screening assessment in clinical settings.

**Supplementary Information:**

The online version contains supplementary material available at 10.1186/s12877-022-03376-x.

## Introduction

Frailty is a recognized health state of elevated susceptibility, hypothesized to emerge from physiologic declines, to adverse health outcomes when encountering a stressor event [1, 2]. It is conceptualized to be syndromic and distinct from disability and comorbidity [3]. Currently, about 15% of non-institutionalized older adults living in the United States are frail [4]; global estimates range up to 27.3% [5].

The physical frailty phenotype (PFP) is a commonly used frailty assessment [6] developed to operationalize the biologic syndrome of frailty [1]. The PFP includes both objective and self-reported measures: objective weakness (grip strength) and slowness (walking speed), unintentional weight loss, and self-reported exhaustion and low activity [1, 7]. A review of modifications to the PFP found that researchers frequently substitute self-reported questions for the objective tests when not available in existing data sets or difficult to collect in settings with limited resources [8–11]. However, the agreement between self-reported and objective measures of walking speed and grip strength and its impact on the overall frailty diagnosis are often not tested.

Previous studies of self-reported versions of the PFP have often used single questions about current physical function / functional difficulty to replace each of the objective measures [12–14]. One study used a set of questions about current functional abilities [15]; another used questions regarding changes in physical function over time [16]. Three studies reported information on agreement between objective and self-reported measures [15–17]. No studies to date have tested multiple self-report substitutions using questions that ask about current function and changes in function. Therefore, a self-reported version of the PFP that has been validated with the standard phenotype and its performance measures could serve to foster frailty detection that is more accurate relative to the physical frailty assessment in diverse clinical settings.

In this cross-sectional study, we examined the agreement between the standard PFP and versions of a self-reported phenotype where objective measures were replaced by subjective questions. Given that the frailty syndrome is conceptually distinct from disability and related to declines in physiologic reserve [1, 3], we hypothesized that: 1) self-reported questions that are used to determine current difficulties in physical function would have lower agreement and worse diagnostic accuracy than change-based questions when compared to the objective measures in the frailty phenotype; and 2) agreement and diagnostic accuracy of self-reported and objective measures would be affected by: a) using multiple self-report questions instead of a single question for slowness and weakness criteria; and b) adding follow-up questions related to severity of difficulty. We aimed to develop a self-reported assessment, with high agreement to the standard PFP, for identifying frail versus non or pre-frail older adults in order to facilitate the detection of frailty in settings where performance testing is less feasible.

## Methods

### Subjects

Study participants were recruited to the “Registry of Older Adults Who May Be Willing to Participate in Research” (IRB# NA_00013162) on the Johns Hopkins Bayview Medical Campus. It is comprised of community-dwelling adults, aged 65 years or older, living in the Baltimore Metropolitan area and recruited from a Johns Hopkins outpatient clinic, off-site educational events, or responses to newsprint and online advertisements. Participants must not have an advanced illness with < 6-month life expectancy. Once consented, demographic and health-related information is collected and participants undergo a frailty assessment protocol by trained personnel. A set of self-reported measures related to walking speed and grip strength (described below) was collected on all registry participants between December 2016 and July 2019. The Johns Hopkins Medicine Institutional Review Board approved this study and analyses.

### Self-reported questions

We used three subjective questions on slowness [18–20] and three on weakness [21–23] that have previously been studied for agreement with objective walking speed and grip strength tests, respectively. This selection was based on a literature review of studies that used these walking and grip measures; we selected a range of self-report measures in order to test agreement alone and in combinations. See Table 1 for the exact wording of the questions used.

- For walking speed, the participants met the slowness criterion if they responded that: *1) they walk very slow or are unable to walk in comparison to the walking speed of their peers; 2) walking a quarter mile is difficult; or 3) they have more difficulty crossing an intersection in a timely manner compared to age 60.* Question 3 served as our dynamic, change-based question for self-reported walking speed.
- For grip strength, the participants met the weakness criterion if they responded that: *4) carrying groceries is difficult; 5) opening jars is difficult; or 6) lifting an object once with slight difficulty soon becomes strenuous if they attempt to lift it repeatedly.* Question 6 served as our dynamic, change-based question for self-reported grip strength.

**Table 1:** Questions for the slowness and weakness criteria in the self-reported physical frailty phenotypes

Criterion	Question Type	Self-reported Questions	Criterion met:	Severity criteria met:
Self-reported Walking Speed^18−20^	Static^a^	1. *"Which of the following best describes your walking speed compared to the walking speed of your peers?"* a) unable to walk; b) very slow; c) stroll at an easy pace; d) normal; e) fairly brisk; f) fast	Answer “a” or “b”	n/a
	Static	2. *“Do you have difficulty walking 1/4 mile (2–3 blocks)?”* Yes or No *If yes, is this task* a) somewhat difficult; b) moderately difficult; or c) very difficult?	Answer “Yes”	Answer “Yes”, and report that this task is: “b) moderately difficult” or “c) very difficult”
	Dynamic^b^	3. *“Compared to age 60, it is more challenging for you to cross an intersection safely because of your walking speed?”* Yes or No *If yes, is it* a) slightly more difficult; b) moderately more difficult; or c) extremely more difficult?	Answer “Yes”	Answer “Yes”, and report that this task is: “b) moderately difficult” or “c) very difficult”
Self-reported Grip Strength^21−23^	Static	4. *"Do you have difficulty lifting or carrying groceries?"* Yes or No *If yes, is this task* a) somewhat difficult; b) moderately difficult; or c) very difficult?	Answer “Yes”	Answer “Yes”, and report: “b) moderately difficult” or “c) very difficult”
	Static	5. *"Do you have difficulty with opening jars?”* Yes or No *If yes, is this task* a) somewhat difficult; b) moderately difficult; or c) very difficult?	Answer “Yes”	Answer “Yes”, and report: “b) moderately difficult” or “c) very difficult”
	Dynamic	6. *"Does an object that you lift once with slight difficulty soon become strenuous when you attempt to lift it repeatedly?"* Yes or No	Answer “Yes.”	n/a

^a^Current function

^b^Change in function

Refs:[18–23]

We examined the agreement between these individual questions (separately and in combination, i.e., a positive response on one or more questions) with their corresponding objective performance measures.

Additionally, we examined agreement when applying the severity of difficulty (mild, moderate, or extreme) as a cutoff for meeting the criteria. Questions 2–5 included follow-up questions regarding level of difficulty. Only those who reported moderate or extreme difficulty met the criteria in this scenario.

### Standard physical frailty phenotype (PFP)

A standard PFP assessment was performed [1, 7]. In brief, we assessed 1) slowness measured by walking speed over 4 m (average of two trials); 2) weakness measured by grip strength in dominant had using a hydraulic dynamometer (max of three trials); 3) shrinking measured by unintentional weight loss in the past year (loss of greater than 5% of body weight, or current body mass index < 18.5 kg/m2); 4) low activity measured by kilocalorie outputs based on algorithms from 6 self-reported questions about activity in the past two weeks; 5) exhaustion measured by positive responses to any of 3 questions about weakness, tiredness and energy level. See Appendix 1 for detailed information on frailty criteria and cut-offs. Participants were defined as frail if three or more of the five criteria were present; pre-frail if one or two criteria were present; and non-frail if no criteria were present. The dichotomized version of the PFP combined the pre-frail and the non-frail groups.

### Self-reported physical frailty phenotype

We developed three self-reported PFPs by using self-reported questions described above in place of the objective walking speed and grip strength tests:

1. A self-reported PFP that included solely “static” questions – meaning questions about current function or functional difficulty; if the response was met for questions 1 or 2 for slowness or for questions 4 or 5 for weakness, the participant was frail for that criterion.
2. A self-reported PFP that included solely “dynamic” questions – meaning questions about changes in functional ability over time; if the response was met for question 3 for slowness or for question 6 for weakness, the participant was frail for that criterion.
3. A self-reported PFP that sought to maximize agreement as well as diagnostic accuracy, specifically sensitivity and negative predictive value (NPV), with the objective measure, to be described in the results.

### Other covariates

Demographic information (age, gender, race, marital status, highest level of education completed) and health-related information (number of diseases, number of falls in past years, history of depression/anxiety) were obtained using a standardized questionnaire. Height and weight were measured to calculate BMI.

### Statistical analysis

We calculated percent agreement by summing the number of values in agreement between objective and self-report measures and dividing by the study N. Inter-rater reliability measured by Cohen’s Kappa coefficient was used to determine the agreement, accounting for chance, between objective and self-reported measures, and between standard and self-reported phenotype instruments [24, 25]. Kappa coefficients were interpreted as follows: 0.01–0.20 as slight agreement; 0.21–0.40 as fair; 0.41–0.60 as moderate; 0.61–0.80 as substantial; and 0.81–1.00 as almost perfect. [24, 26] We calculated 95% confidence intervals for all Kappa scores.

To assess the validity of self-reported measures in comparison with corresponding objective tests, we calculated sensitivity, specificity, positive predictive value (PPV), and negative predictive value (NPV). Sensitivity refers to a screening test’s ability to accurately identify a condition among those who actually have the condition as determined by a reference standard) [27]. Specificity is a test’s ability to accurately identify those who do not have a condition among those who truly do not have it [27]. PPV is a test’s ability to correctly identify those who have the condition among those with a positive screening test [27]. NPV is the ability of a test to correctly identify those who do not have the condition among those with a negative screening test [27]. We decided to prioritize sensitivity and NPV a priori in order to avoid false negatives. We performed the same validity calculations for the dichotomized (frail versus pre/non-frail) self-reported PFP compared with a dichotomized standard phenotype.

## Results

One-hundred-eighty-two registry participants completed the standard frailty phenotype assessment and a self-report questionnaire of walking and grip related questions. As shown in Table 2, the participants were 65 to 98 years of age, with a mean age of 75.5 years (SD = 8.1). The population was 64.3% female and 74.7% Caucasian, with 56% having completed their college education or higher, and 45.6% married at time of assessment. Using the standard PFP, 12.1% were categorized as frail, versus 44.5% pre-frail or 43.4% non-frail. 28.6% met criteria for slowness and 39% met criteria for weakness.

**Table 2:** Summary of the descriptive characteristics of the study population

Characteristic	Value
Sample Size, n	182
Age, mean years (range, standard deviation)	75.5 (65–98, 8.1)
Gender, % Female	64.3%
Race	
% Caucasian	74.7%
% African American	19.2%
% Other (American Indian, Asian, Hispanic, Mixed, Other)	6.0%
Education	
% College/some grad/grad school	56.0%
% High school /some college/vocational school	39.6%
% Less than high school	3.8%
Marital Status^a^	
% Married	45.6%
% Widowed	19.2%
% Divorced / Separated	18.6%
% Single	12.1%
Health Information	
Body Mass Index, mean (standard deviation, SD)	28.4 (6.6)
Number of diseases (self-report), mean (SD)^b^	3.5 (1.9)
Number of falls in past 2 years, mean (SD)^b^	0.88 (1.7)
Reported depression/anxiety (self-report; % Yes)^b^	26.6%
Usual gait speed, m/s, mean (SD)	0.86 (0.23)
Maximal grip strength, kg, mean (SD)	25.8 (8.9)
Standard Physical Frailty Phenotype (PFP)	
% Frail	12.1%
% Pre-Frail	44.5%
% Non-Frail	43.4%
Components of Standard PFP	
% Exhaustion	12.1%
% Low physical activity	14.3%
% Slowness	28.6%
% Weakness	39.0%
% Weight Loss	3.8%
Self-Report PFP using only static self-report questions	
% Frail	11.5%
% Pre-Frail	58.2%
% Non-Frail	30.2%
Components of Self-Report PFP using static self-report questions	
% Slowness	26.5%
% Weakness	56.6%
Self-Report PFP using only dynamic self-report questions	
% Frail	9.9%
% Pre-Frail	46.7%
% Non-Frail	43.4%
Components of Self-Report PFP using dynamic self-report questions	
% Slowness	32.8%
% Weakness	32.6%
Self-Report PFP using all self-report questions	
% Frail	13.7%
% Pre-Frail	62.1%
% Non-Frail	24.2%
Components of Self-Report PFP using all self-report questions	
% Slowness	39.8%
% Weakness	65.2%

^a^8 participants missing marital status

^b^12 participants missing information on diseases, falls, depression/anxiety

### Objective versus self-reported walking speed and grip strength assessment

The agreement between measured walking speed and the individual self-reported slowness questions was fair for Question 1 (Kappa(k) = 0.34), and moderate for Question 2 (k = 0.54) and Question 3 (k = 0.47). By combining questions, including using all three questions, we found concordance still within the moderate range (k = 0.51–0.56). Highest sensitivity and NPV were found when comparing walking speed with a combination of all questions; highest specificity and PPV were found using Question 1. See Table 3.

**Table 3:** Summary of agreement and predictive accuracy statistics for objective vs self-report measures

Measures Compared	Percent Agreement	Kappa Coefficient (95%CI)	Sensitivity	Specificity	PPV	NPV
Objective Slowness vs. Walking Question 1 (Static)	77.9%	0.34 (0.16, 0.52)	31%	**97%**	**80%**	78%
Objective Slowness vs. Walking Question 2 (Static)	**82.4%**	0.54 (0.40, 0.69)	60%	92%	74%	85%
Objective Slowness vs. Walking Questions 1 and 2 Combined (Static)	82.3	**0.56 (0.38, 0.74)**	65%	89%	71%	86%
Objective Slowness vs. Walking Question 3 (Dynamic)	77.5%	0.47 (0.33, 0.61)	67%	82%	59%	86%
Objective Slowness vs. Walking Questions 1–3 Combined	77.9%	0.51 (0.38, 0.65)	**81%**	77%	58%	**91%**
Objective Weakness vs. Grip Question 4 (Static)	63.2%	0.16 (-0.00, 0.32)	29%	**85%**	**57%**	65%
Objective Weakness vs. Grip Question 5 (Static)	56.0%	0.13 (-0.01, 0.27)	61%	53%	46%	67%
Objective Weakness vs. Grip Questions 4 and 5 Combined (Static)	55.5%	0.13 (-0.01, 0.27)	65%	49%	46%	68%
Objective Weakness vs. Grip Question 6 (Dynamic)	**63.5%**	**0.21 (0.05, 0.36)**	45%	75%	54%	68%
Objective Weakness vs. Grip Questions 4–6 Combined	51.9%	0.10 (-0.04, 0.23)	**72%**	39%	43%	68%

Bolded text = highest value per column, per category (slowness, weakness)

*Abbreviations*: *95%CI* 95% confidence interval, *PPV* Positive Predictive Value, *NPV* Negative Predictive Value

The agreement between measured grip strength and the self-reported weakness questions was slight for Question 4 (k = 0.16) and Question 5 (k = 0.13), and fair for Question 6 (k = 0.21). By combining questions, including using all questions, we found slight agreement (k = 0.10–0.13). The highest sensitivity was found when comparing grip strength with a combination of all questions (72%), and the highest specificity (85%) and PPV (57%) with Question 4. Comparable values for NPV were found using individual or combined questions (65–68%). See Table 3. Appendix 2 provides tabulations.

We also explored agreement and accuracy when including measures of severity (e.g., moderate or extreme difficulty) in the self-reported slowness and weakness questions. The findings were similar to the results without accounting for the severity of difficulty (see Appendix 3).

### Standard versus self-reported frailty assessments

In comparison to the prevalence of frailty assessed with the standard PFP (see Table 2), the self-reported PFP with only static self-report items substituted for the slowness and weakness criteria found 11.5% frail, versus 58.2% pre-frail or 30.2% non-frail. The self-reported PFP with only dynamic self-report items substituted for the slowness and weakness criteria found 9.9% were frail, versus 46.7% pre-frail or 43.4% non-frail. The self-reported PFP with all self-report items substituted for the slowness and weakness criteria found 13.7% frail, versus 62.1% pre-frail or 24.2% non-frail. See tabulations in Appendix 2c-e.

Comparing the dichotomized (frail vs non/pre-frail) standard PFP to the self-reported PFP, substantial agreement was found across the different self-reported PFPs (k = 0.76–0.78), with confidence intervals remaining in the substantial range. Highest sensitivity (86.4%) was found in the version using all self-reported questions for slowness and weakness criteria. All versions of the dichotomized self-reported PFP had high specificity (96.3–98.8%) and high NPV (96.3–98.7%). The greatest PPV (88.9%) was found in the version using only dynamic self-reported questions. See Table 4 for further details.

**Table 4:** Summary of agreement and predictive accuracy statistics for objective vs self-report physical frailty phenotypes in the study population

Measures Compared	Percent Agreement	Kappa Coefficient (95%CI)	Sensitivity	Specificity	PPV	NPV
**Dichotomized Scoring**^**a**^						
Standard PFP vs. Self-reported PFP—Static Questions	95.0%	0.76 (0.61, 0.91)	77.3%	97.5%	81%	96.9%
Standard PFP vs. Self-reported PFP—Dynamic Questions	**95.6%**	0.78 (0.62, 0.93)	73%	**98.8%**	**88.9%**	96.3%
Standard PFP vs. Self-reported PFP—All Questions	95.0%	**0.78 (0.64, 0.92)**	**86.4%**	96.3%	76%	**98.7%**
**Three-level Scoring**^**b**^						
Standard PFP vs. Self-reported PFP—Static Questions	59.9%	0.33 (0.21, 0.45)	n/a	n/a	n/a	n/a
Standard PFP vs. Self-reported PFP—Dynamic Questions	**70.3%**	**0.50 (0.39, 0.61)**	n/a	n/a	n/a	n/a
Standard PFP vs. Self-reported PFP—All Questions	60.4%	0.35 (0.23, 0.46)	n/a	n/a	n/a	n/a

Measures of slowness and weakness in the self-reported phenotypes are from self-report questions only

Bolded text = highest value per column, per category (dichotomized, three-level)

*Abbreviations*: *PFP* Physical frailty phenotype, *95%CI* 95% confidence interval, *PPV* Positive Predictive Value, *NPV* Negative Predictive Value

^a^ Phenotypes dichotomized by frail (3 or more criteria met) and pre/non-frail (0–2 criteria met)

^b^ Phenotypes have three levels: frail (3 or more criteria met); pre-frail (1–2 criteria met) or non-frail (0 criteria met)

Comparing agreement between the three-level (frail vs pre-frail vs non-frail) standard PFP and the self-reported PFP – where only the static self-report questions for walking speed and grip strength were combined to determine slowness and weakness, respectively – we found fair agreement (k = 0.33). See Table 4. When dynamic (change-based) questions alone were included for slowness and weakness in the three-level self-reported PFP, the agreement increased to moderate (k = 0.50). Similar to the static-only questions, we found fair agreement (k = 0.35) when we used all questions for slowness and weakness criteria in the three-level self-reported PFP.

## Discussion

When compared to the dichotomized (frail vs pre/non-frail) standard PFP, we defined a self-reported PFP with substantial agreement (k = 0.78), high sensitivity (84.6%), and high NPV (98.7%). This self-reported PFP combined all self-reported questions per the slowness and weakness criteria. However, in comparison to the three-level (frail vs pre-frail vs non-frail) standard PFP, the three-level self-reported versions in this study had only fair to moderate agreement (k = 0.33–0.50). We found that including more self-report items led to increased sensitivity for frailty (vs. non-frail) detection while maintaining similar levels of NPV. The importance of identifying and treating frailty early in its course, as well as the relative ease of performing more objective tests for confirmation, if needed, without harm to patients even if frailty is not present, makes reducing the false negatives imperative [27].

In comparison to objective measures of slowness and weakness in the standard PFP, self-reported walking questions had fair to moderate agreement with objective slowness, while self-reported grip/hand strength questions had slight to borderline fair agreement with objective weakness. This discrepancy may be due to the specific self-reported questions included in this study. The combination of multiple self-reported questions led to slightly improved Kappa agreement over the individual questions for slowness only. However, including multiple self-reported items had a marked impact on improving sensitivity relative to NPV. Static self-reported questions commonly used to determine difficulties in physical function (e.g., difficulty walking ¼ mile or lifting groceries) had fair to moderate agreement with objective slowness but only slight agreement with objective weakness. Change-based dynamic questions showed moderate agreement for walking speed but only fair agreement for grip strength. Using self-reported questions that included severity, we found minimal change in agreement scores, but both sensitivity and NPV decreased. Therefore, self-reported measures of slowness and weakness that include degrees of difficulty do not appear to improve efforts to minimize false negatives (high sensitivity) and to maximize true negatives (high NPV) [27].

Our work provides novel information about agreement and diagnostic accuracy between standard and self-reported frailty phenotypes, and expands upon on previous studies that have examined agreement between subjective and objective measurements for weakness and slowness. A 2018 study reported observed agreement of 71.1% and k = 0.55 between a self-reported (including four static slowness questions and two weakness questions) and standard three-level frailty phenotype [15], which aligns with the results in our study (agreement = 70.3%; k = 0.50) when we substituted dynamic self-report questions for slowness and weakness criteria. Nunes and colleagues found that self-reported decline in walking speed over the past year, compared to objective walking speed, had 79% sensitivity, 31% specificity, 56% PPV, and 57% NPV [16], and self-reported decreased strength / increased weakness in the past year, in comparison to measured grip strength, had 78% sensitivity, 35% specificity, 48% PPV, and 70% NPV. In our cross-sectional analyses, using the same validity tests, our combined self-reported slowness measures showed equal or higher values for all tests, and our combined self-reported weakness measures showed comparable values. Additionally, in a study of hemodialysis patients, Johansen et al. substituted the self-reported physical function score from the Short-Form 36 questionnaire for the objective slowness and weakness criteria in a self-reported PFP [17].

A critical consideration is whether self-reported information can fully equate with performance measures [28, 29]. Several studies, including our present study, have shown varying levels of agreement between self-reported and objective measures of walking speed and grip strength [29–33]. A systematic review reported that studies that compared self-reported measures and performance measures of the same construct (e.g., functional limitation with functional limitation) showed higher levels of correlation than studies that compared different constructs (functional limitation compared to disability measures) [29]. Despite these issues, self-report items remain relevant given that time and resources required for objective data capture and analyses are often less clinically feasible. Also, the limits of healthcare and research activities in scenarios such as the COVID-19 pandemic provide for timely consideration of remote assessment options.

We tested agreement between our new self-reported frailty assessment and the PFP. We focused on this comparison due to the physiological basis of frailty assessment using the PFP and the potential avenues for prevention and treatment this basis offers [34]. We further reason that using the PFP to define frailty syndrome holds potential to allow researchers and clinicians to a) identify drivers of elevated risk that in turn will suggest strategies to lessen risk, and b) research underlying etiology such that we may eventually be able to prevent or delay frailty onset. Thus, we assert that close approximation of the self-reported PFP to the standard version is important. Though we did not compare with existing self-report frailty measures such as the FRAIL Scale or Clinical Frailty Scale [35, 36], we note the previous analyses have found a lack of agreement in frailty categorization between these scales and the PFP [37].

Regarding existing self-report measures, we did not include the FRAIL Scale in our main study for two main reasons: 1) we view it as conceptually distinct from physical frailty due the inclusion of multimorbidity as one of its criteria; 2) data to calculate the FRAIL scale was available only for a subset of our study population. However, we did analyze the agreement between the standard PFP and the self-reported version of the PFP, and the agreement between the standard PFP and the FRAIL scale, in a subset of our study population (*N* = 166 participants with available data, out of 182 participants total). We found fair agreement (0.36) between the standard PFP and the FRAIL scale when assessing frail vs non-frail status, which is consistent with previous studies. In these same subset analyses, we found substantial agreement (k = 0.76) for the self-reported PFP, as well as greater sensitivity, positive predictive value, and negative predictive value for the self-reported PFP. See Appendix 4 for additional details.

Study limitations include a non-representative population of older adults and a relatively small sample size of 182, of which only 22 were frail. Because the participants were enrolled in an aging study registry and typically able to perform the objective tasks, this population may be of better health than the average older adult. In fact, prevalence of frailty in our study is below the U.S. nationally representative average [4]. Related to prevalence, we noted that the improved Kappa agreement between the standard three-level PFP and the dynamic self-reported PFP is likely influenced by their comparable frailty prevalence.

## Conclusions

We found substantial agreement, high specificity, and high negative predictive value with a dichotomous self-reported frailty phenotype when compared to the standard PFP. However, the limitations of our study population and sample size are challenges to the generalizability of these findings. We believe a self-report PFP can serve as a useful screening assessment; those who are frail based on a self-reported PFP could then be tested with objective measures to verify frailty status. Future studies are needed in broader populations to examine the agreement and accuracy of these type of self-reported physical frailty phenotypes.

## Supplementary Information

**Additional file 1: Appendix 1.** Criteria used to assess frailty status in the standard frailty phenotype. **Appendix 2.** Agreement using Cohen’s Kappa coefficient statistic. **Appendix 2a.** Agreement between the objective slowness criterion and self-reported slowness questions. **Appendix2b.** Agreement between the objective weakness criterion and self-reported weakness questions. **Appendix 2c.** Agreement between the standard frailty phenotype and self-Reported frailty phenotype with static questions. **Appendix 2d.** Agreement between the standard frailty phenotype and self-reported frailty phenotype with dynamic questions. **Appendix 2e.** Agreement between the standard frailty phenotype and self-reported frailty phenotype with all questions. **Appendix 3.** Summary of agreement and predictive accuracy statistics for objective vs self-report measures with Severity Criteria. **Appendix 4. **Summary of agreement and predictive accuracy statistics for objective vs self-report physical frailty phenotype (PFP) and objective PFP vs FRAIL scale in a subset (N=166) of the study population.

## Data Availability

The datasets used and/or analyzed during the current study are available from the corresponding author on reasonable request.

## Abbreviations

BMI: Body Mass Index
NPV: Negative Predictive Value
PFP: Physical Frailty Phenotype
PPV: Positive Predictive Value
