# Supplementary material for: Agreement between standard and self-reported assessments of physical frailty syndrome and its components in a registry of community-dwelling older adults
Source: BMC Geriatr. 2022 Aug 25;22:705. doi: 10.1186/s12877-022-03376-x (PMC9403951; doi:10.1186/s12877-022-03376-x)
Supplement: Supplementary file 1 — Additional file 1: Appendix 1. Criteria used to assess frailty status in the standard frailty phenotype. Appendix 2. Agreement using Cohen’s Kappa coefficient statistic. Appendix 2a. Agreement between the objective slowness criterion and self-reported slowness questions. Appendix2b. Agreement between the objective weakness criterion and self-reported weakness questions. Appendix 2c. Agreement between the standard frailty phenotype and self-Reported frailty phenotype with static questions. Appendix 2d. Agreement between the standard frailty phenotype and self-reported frailty phenotype with dynamic questions. Appendix 2e. Agreement between the standard frailty phenotype and self-reported frailty phenotype with all questions. Appendix 3. Summary of agreement and predictive accuracy statistics for objective vs self-report measures with Severity Criteria. Appendix 4. Summary of agreement and predictive accuracy statistics for objective vs self-report physical frailty phenotype (PFP) and objective PFP vs FRAIL scale in a subset (N=166) of the study population. [file 12877_2022_3376_MOESM1_ESM.docx]

**Appendix 1: Criteria used to Assess Frailty Status in the Standard Frailty Phenotype.**

**Scoring: ≥3/5 criteria indicates frailty; 1-2 indicates pre-frailty; 0 indicates non-frail.**

| **Criterion** | **Meets criterion for frailty if** | |
| --- | --- | --- |
| **Weight loss** | Lost >5% body weight unintentionally in last year, or BMI <18.5kg/m^2^  Equipment: scale for body weight; stadiometer for height. | |
| **Exhaustion** | Felt unusually tired or unusually weak ‘all of the time’ or ‘most of the time’ or reported energy level was ≤3. Determined by:  *1.a. “In the past month, on the average, have you been feeling unusually tired during the day?”*    Yes □ No □ Refused □ Don’t Know □  *1.b. “If yes, have you been feeling unusually tired:”*   - All of the time - Most of the time - Some of the time - Refused / Don’t Know   *2.a. “In the past month, on the average, have you felt unusually weak?”*  Yes □ No □ Refused □ Don’t Know □ *2.b.” If yes, have you been feeling weak:”*   - All of the time - Most of the time - Some of the time - Refused / Don’t Know   *3. “Using the scale below, please rate your usual energy level on a scale from 0 to 10 where 0 is no energy and 10 is the most energy that you have ever had. Please give a number between 0 and 10 that describes your usual energy level while awake in the last month?”* Energy Level: ____ | |
| **Slowness (walking speed over 4 meters)** | **Men**  ≤.65m/s for height ≤173 cm (68 inches)  ≤.76m/s for height >173 cm (68 inches) | **Women**  ≤.65m/s for height ≤159cm (63 inches)  ≤.76m/s for height >159cm (63 inches) |
|  | Equipment: 4-meter course, a stopwatch.  Participant walks 4-meter length twice at his or her usual pace. Use average of 2 trials. | |
| **Low Activity Level** | **Men:** <128 kcal of physical expenditure on activity scale per week (6 items^a^) | **Women:** <90 kcal of physical expenditure on activity scale per week (6 items^a^) |
| **Weakness (grip strength, maximal score with dominant hand)** | **Men**  ≤29 kg for BMI ≤24  ≤30 kg for BMI 24.1–26  ≤30 kg for BMI 26.1–28  ≤32 kg for BMI >28 | **Women**  ≤17 kg for BMI ≤23  ≤17.3 kg for BMI 23.1–26  ≤18 kg for BMI 26.1–29  ≤21 kg for BMI >29 |
|  | Equipment: (Jamar) hand dynamometer.  Participant attempts to squeeze the dynamometer maximally 3 times with the dominant hand. | |
| ^a^ Physical activity is based on modified Minnesota Leisure Time Activities Questionnaire, asking about walking (w = 3.5), strenuous household chores (w = 4.5), strenuous outdoor chores (w = 4.5), dancing (w = 5.5), bowling (w = 3.0), and exercise (w = 4.5). To compute kcals expended per week, use the formula: Kcals (Kilocalories / week) = w * Frequency (sessions per week) * Duration per session (minutes) * Body Weight (kg)/60, where w is the task-specific MET intensity score. | | |
| References: Fried LP et al. Frailty in older adults: evidence for a phenotype. J Gerontol Med Sci. 2001. Bandeen-Roche et al, Phenotype of Frailty: Characterization in the Women’s Health and Aging Studies. J Gerontol Med Sci. 2006. See also: <https://hopkinsfrailtyassessment.org> | | |

**Appendix 2: Agreement using Cohen’s Kappa coefficient statistic**

**Appendix 2a. Agreement between the Objective Slowness Criterion and Self-reported Slowness Questions.**

|  |  | **Objective Slowness** |  |
| --- | --- | --- | --- |
| **Self-report Walking 1 (static)** | Frail | Non | Total |
| Frail | 16 | 4 | 20 |
| Non | 36 | 125 | 161 |
| Total | 52 | 129 | 181 |
|  |  |  |  |
| Agreement | 16 | 125 | 141 |
| By Chance | 5.745856 | 114.7459 | 120.4917 |
|  |  |  |  |
| Kappa | **0.338934** |  |  |
| Note: excluded 1 participant who declined to answer self-report. | | | |
|  |  |  |  |
|  |  | **Objective Slowness** |  |
| **Self-report Walking 2 (static)** | Frail | Non | Total |
| Frail | 31 | 11 | 42 |
| Non | 21 | 119 | 140 |
| Total | 52 | 130 | 182 |
|  |  |  |  |
| Agreement | 31 | 119 | 150 |
| By Chance | 12 | 100 | 112 |
|  |  |  |  |
| Kappa | **0.542857** |  |  |
|  |  |  |  |
|  |  | **Objective Slowness** |  |
| **Self-report Walking 3 (dynamic)** | Frail | Non | Total |
| Frail | 35 | 24 | 59 |
| Non | 17 | 106 | 123 |
| Total | 52 | 130 | 182 |
|  |  |  |  |
| Agreement | 35 | 106 | 141 |
| By Chance | 16.85714 | 87.85714 | 104.7143 |
|  |  |  |  |
| Kappa | **0.469501** |  |  |
|  |  |  |  |
|  |  | **Objective Slowness** |  |
| **Self-report Walking ALL (static & dynamic)** | Frail | Non | Total |
| Frail | 41 | 29 | 70 |
| Non | 11 | 101 | 112 |
| Total | 52 | 130 | 182 |
|  |  |  |  |
| Agreement | 41 | 101 | 142 |
| By Chance | 20 | 80 | 100 |
|  |  |  |  |
| Kappa | **0.512195** |  |  |

|  |  | **Objective Slowness** |  |  |  |
| --- | --- | --- | --- | --- | --- |
| **Self-report Walking 1 (static) and 2 (static)** | Frail | Non | Total |  |  |
| Frail | 34 | 14 | 48 |  |  |
| Non | 18 | 115 | 133 |  |  |
| Total | 52 | 129 | 181 |  |  |
|  |  |  |  |  |  |
| Agreement | 34 | 115 | 149 |  |  |
| By Chance | 13.79006 | 94.79006 | 108.5801 |  |  |
|  |  |  |  |  |  |
| Kappa | **0.558132** |  |  |  |  |
| Note: excluded 1 participant who declined to answer self-report Q1. | | | | | |

|  |  | **Objective Slowness** |  |
| --- | --- | --- | --- |
| **Self-report Walking 1 (static) and 3 (dynamic)** | Frail | Non | Total |
| Frail | 39 | 26 | 65 |
| Non | 13 | 103 | 116 |
| Total | 52 | 129 | 181 |
|  |  |  |  |
| Agreement | 39 | 103 | 142 |
| By Chance | 18.67403 | 82.67403 | 101.3481 |
|  |  |  |  |
| Kappa | **0.51037** |  |  |

|  |  | **Objective Slowness** |  |
| --- | --- | --- | --- |
| **Self-report Walking 2 (static) and 3 (dynamic)** | Frail | Non | Total |
| Frail | 41 | 28 | 69 |
| Non | 11 | 102 | 113 |
| Total | 52 | 130 | 182 |
|  |  |  |  |
| Agreement | 41 | 102 | 143 |
| By Chance | 19.71429 | 80.71429 | 100.4286 |
|  |  |  |  |
| Kappa | **0.521891** |  |  |
|  |  |  |  |

**Appendix 2b. Agreement between the Objective Weakness Criterion and Self-reported Weakness Questions.**

|  |  | **Objective Weakness** |  |
| --- | --- | --- | --- |
| **Self-report Grip 4 (static)** | Frail | Non | Total |
| Frail | 21 | 16 | 37 |
| Non | 51 | 94 | 145 |
| Total | 72 | 110 | 182 |
|  |  |  |  |
| Agreement | 21 | 94 | 115 |
| By Chance | 14.63736 | 87.63736 | 102.2747 |
|  |  |  |  |
| Kappa | **0.159614** |  |  |
|  |  |  |  |
|  |  | **Objective Weakness** |  |
| **Self-report Grip 5 (static)** | Frail | Non | Total |
| Frail | 44 | 52 | 96 |
| Non | 28 | 58 | 86 |
| Total | 72 | 110 | 182 |
|  |  |  |  |
| Agreement | 44 | 58 | 102 |
| By Chance | 37.97802 | 51.97802 | 89.95604 |
|  |  |  |  |
| Kappa | **0.13085** |  |  |
|  |  |  |  |
|  |  | **Objective Weakness** |  |
| **Self-report Grip 6 (dynamic)** | Frail | Non | Total |
| Frail | 32 | 27 | 59 |
| Non | 39 | 83 | 122 |
| Total | 71 | 110 | 181 |
|  |  |  |  |
| Agreement | 32 | 83 | 115 |
| By Chance | 23.14365 | 74.14365 | 97.28729 |
|  |  |  |  |
| Kappa | **0.211589** |  |  |
| Note: excluded 1 participant who declined to answer self-report. | | | |
|  |  |  |  |
|  |  | **Objective Weakness** |  |
| **Self-report Grip ALL (static and dynamic)** | Frail | Non | Total |
| Frail | 51 | 67 | 118 |
| Non | 20 | 43 | 63 |
| Total | 71 | 110 | 181 |
|  |  |  |  |
| Agreement | 51 | 43 | 94 |
| By Chance | 46.28729 | 38.28729 | 84.57459 |
|  |  |  |  |
| Kappa | **0.097748** |  |  |

|  |  | **Objective Weakness** |  |
| --- | --- | --- | --- |
| **Self-report Grip 4 (static) and 5 (static)** | Frail | Non | Total |
| Frail | 47 | 56 | 103 |
| Non | 25 | 54 | 79 |
| Total | 72 | 110 | 182 |
|  |  |  |  |
| Agreement | 47 | 54 | 101 |
| By Chance | 40.74725 | 47.74725 | 88.49451 |
|  |  |  |  |
| Kappa | **0.133741** |  |  |
|  |  |  |  |
|  |  | **Objective Weakness** |  |
| **Self-report Grip 4 (static) and 6 (dynamic)** | Frail | Non | Total |
| Frail | 37 | 36 | 73 |
| Non | 34 | 74 | 108 |
| Total | 71 | 110 | 181 |
|  |  |  |  |
| Agreement | 37 | 74 | 111 |
| By Chance | 28.63536 | 65.63536 | 94.27072 |
|  |  |  |  |
| Kappa | **0.192891** |  |  |
| Note: excluded 1 participant who declined to answer self-report 6. | | | |
|  | | | |
|  |  | **Objective Weakness** |  |
| **Self-report Grip 5 (static) and 6 (dynamic)** | Frail | Non | Total |
| Frail | 50 | 65 | 115 |
| Non | 21 | 45 | 66 |
| Total | 71 | 110 | 181 |
|  |  |  |  |
| Agreement | 50 | 45 | 95 |
| By Chance | 45.1105 | 40.1105 | 85.22099 |
|  |  |  |  |
| Kappa | **0.1021** |  |  |

Note: excluded 1 participant who declined to answer self-report 6.

**Appendix 2c. Agreement between the Standard Frailty Phenotype and Self-Reported Frailty Phenotype with Static questions***

|  |  |  |  |  |
| --- | --- | --- | --- | --- |
|  |  |  |  |  |
| **DICHOTOMOUS** | **Standard Frailty Phenotype** | | |  |
| **Self-Report Frailty Phenotype*** | Frail |  | Non/Pre | Total |
| Frail | 17 |  | 4 | 21 |
|  |  |  |  |  |
| Non/Pre | 5 |  | 156 | 161 |
| Total | 22 |  | 160 | 182 |
|  |  |  |  |  |
| Agreement | 17 |  | 156 | 173 |
| By Chance | 2.538462 |  | 141.5385 | 144.0769 |
|  |  |  |  |  |
| Kappa | **0.762677** |  |  |  |

| **THREE LEVEL** | **Standard Frailty Phenotype** | | |  |
| --- | --- | --- | --- | --- |
| **Self-Report Frailty Phenotype*** | Frail | Pre | Non | Total |
| Frail | 17 | 4 | 0 | 21 |
| Pre | 5 | 56 | 45 | 106 |
| Non | 0 | 19 | 36 | 55 |
| Total | 22 | 79 | 81 | 182 |
|  |  |  |  |  |
| Agreement | 17 | 56 | 36 | 109 |
| By Chance | 2.538462 | 46.01099 | 24.47802 | 73.02747 |
|  |  |  |  |  |
| Kappa | **0.330106** |  |  |  |

*Self-reported phenotype includes the substitution of static questions (questions 1-2 for slowness, and questions 4-5 for weakness) only for objective measures. See Figure 1.

**Appendix 2d. Agreement between the Standard Frailty Phenotype and Self-Reported Frailty Phenotype with Dynamic questions***

| **DICHOTOMOUS** | **Standard Frailty Phenotype** | | |  |
| --- | --- | --- | --- | --- |
| **Self-Report Frailty Phenotype*** | Frail |  | Non/Pre | Total |
| Frail | 16 |  | 2 | 18 |
|  |  |  |  |  |
| Non/Pre | 6 |  | 158 | 164 |
| Total | 22 |  | 160 | 182 |
|  |  |  |  |  |
| Agreement | 16 |  | 158 | 174 |
| By Chance | 2.175824 |  | 144.1758 | 146.3516 |
|  |  |  |  |  |
| Kappa | **0.775586** |  |  |  |

| **THREE LEVEL** | **Standard Frailty Phenotype** | | |  |
| --- | --- | --- | --- | --- |
| **Self-Report Frailty Phenotype*** | Frail | Pre | Non | Total |
| Frail | 16 | 2 | 0 | 18 |
| Pre | 6 | 55 | 24 | 85 |
| Non | 0 | 22 | 57 | 79 |
| Total | 22 | 79 | 81 | 182 |
|  |  |  |  |  |
| Agreement | 16 | 55 | 57 | 128 |
| By Chance | 2.175824 | 36.8956 | 35.15934 | 74.23077 |
|  |  |  |  |  |
| Kappa | **0.498929** |  |  |  |

*Self-reported phenotype includes the substitution of dynamic questions (question 3 for slowness and question 6 for weakness) only for objective measures. See Figure 1.

**Appendix 2e. Agreement between the Standard Frailty Phenotype and Self-Reported Frailty Phenotype with All questions***

| **DICHOTOMOUS** | **Standard Frailty Phenotype** | | |  |
| --- | --- | --- | --- | --- |
| **Self-Report Frailty Phenotype*** | Frail |  | Non/Pre | Total |
| Frail | 19 |  | 6 | 25 |
|  |  |  |  |  |
| Non/Pre | 3 |  | 154 | 157 |
| Total | 22 |  | 160 | 182 |
|  |  |  |  |  |
| Agreement | 19 |  | 154 | 173 |
| By Chance | 3.021978 |  | 138.021978 | 141.043956 |
|  |  |  |  |  |
| Kappa | **0.780252** |  |  |  |

| **THREE LEVEL** | **Standard Frailty Phenotype** | | |  |
| --- | --- | --- | --- | --- |
| **Self-Report Frailty Phenotype*** | Frail | Pre | Non | Total |
| Frail | 19 | 6 | 0 | 25 |
| Pre | 3 | 60 | 50 | 113 |
| Non | 0 | 13 | 31 | 44 |
| Total | 22 | 79 | 81 | 182 |
|  |  |  |  |  |
| Agreement | 19 | 60 | 31 | 110 |
| By Chance | 3.021978 | 49.04945 | 19.58242 | 71.65385 |
|  |  |  |  |  |
| Kappa | **0.347508** |  |  |  |

*Self-reported phenotype includes the substitution of all questions - static and dynamic (questions 1-3 for slowness, and questions 4-6 for weakness) - for objective measures. See Figure 1.

**Appendix 3: Summary of Agreement and Predictive Accuracy Statistics for Objective vs Self-Report Measures *with Severity Criteria^a^***

| **Measures Compared** | **Percent Agreement (∆)** | **Kappa Coefficient (∆)** | **Sensitivity (∆)** | **Specificity (∆)** | **Positive Predictive Value (∆)** | **Negative Predictive Value (∆)** |
| --- | --- | --- | --- | --- | --- | --- |
| Objective Slowness vs. Walking Question 1 (Static) – *Same* | 77.9% (n/a) | 0.34 (n/a) | 31% (n/a) | **97%** (n/a) | **80%** (n/a) | 78% (n/a) |
| Objective Slowness vs. Walking Question 2 (Static) *with Severity* | 72.9%  (-9.5) | 0.20 (-.34) | 35% (-25) | 95% (+3) | 75% (+1) | 79% (-6) |
| Objective Slowness vs. Walking Questions 1 and 2 (Static combined) *with Severity* | 69.4% (-12.9) | 0.16 (-.40) | 49% (-16) | 93% (+4) | 74% (+3) | 82% (-4) |
| Objective Slowness vs. Walking Question 3 (Dynamic) *with Severity* | 77.3%  (-0.2) | 0.39 (-.06) | 45% (-20) | 90% (+8) | 64% (+6) | 81% (-4) |
| Objective Slowness vs. Walking Questions 1-3 (All Combined) *with Severity* | **81.1%** (+3.1) | **0.53** (+.02) | **67%** (-12) | 87% (+9) | 67% (+8) | **87%** (-3) |
|  |  |  |  |  |  |  |
| Objective Weakness vs. Grip Question 4 (Static) *with Severity* | 62.1%  (-1.1) | 0.08 (-.08) | 13% (-16) | **95% (+10)** | **60%** (+3) | 62% (-3) |
| Objective Weakness vs. Grip Question 5 (Static) *with Severity* | 61.1% (+5.1) | 0.13 (0) | 31% (-30) | 81% (+28) | 51% (+5) | 64% (-5) |
| Objective Weakness vs. Grip Questions 4 and 5 (Static combined) *with Severity* | 60.5% (+5.0) | 0.13 (0) | 37% (-18) | 76% (+27) | 50% (+4) | 65% (-3) |
| Objective Weakness vs. Grip Question 6 (Dynamic) – *Same* | **63.5%** (n/a) | **0.21** (n/a) | 45% (n/a) | 75% (n/a) | 54% (n/a) | **68%** (n/a) |
| Objective Weakness vs. Grip Questions 4-6 (All Combined) *with Severity* | 57.4% (+5.2) | 0.13 (+.03) | **52%** (-20) | 61% (+22) | 46% (+2) | 66% (-2) |

Bolded text = highest value per column, per category. **∆** = change in values between Table 2 (without severity criteria) and Appendix 3 (with severity criteria applied).

^a^Severity cut-off: Reporting level of difficulty of tasks to be moderately or very difficult. See Figure 1.

**Appendix 4:** **Summary of Agreement and Predictive Accuracy Statistics for Objective vs Self-Report Physical Frailty Phenotype (PFP) and Objective PFP vs FRAIL Scale in a Subset (N=166) of the Study Population.**

| **Measures Compared** | **Percent Agreement** | **Kappa Coefficient (95%CI)** | **Sensitivity** | **Specificity** | **PPV** | **NPV** |
| --- | --- | --- | --- | --- | --- | --- |
| **Dichotomized Scoring^a^** |  |  |  |  |  |  |
| Standard PFP vs. Self-reported PFP^c^ | **95.0%** | **0.76 (0.61, 0.91)** | **85.0%** | 95.9% | **73.9%** | **97.9%** |
| Standard PFP vs. FRAIL Scale^d^ | 88.5% | 0.36 (0.09, 0.63) | 35.0% | 95.9% | 53.8% | 91.5% |
|  |  |  |  |  |  |  |
| **Three-level Scoring^b^** |  |  |  |  |  |  |
| Standard PFP vs. Self-reported PFP | 58.4% | **0.32 (0.20, 0.44)** | n/a | n/a | n/a | n/a |
| Standard PFP vs. FRAIL Scale | **59.6%** | 0.30 (0.17, 0.43) | n/a | n/a | n/a | n/a |
|  |  |  |  |  |  |  |

^a^ Phenotypes and FRAIL Scale dichotomized by frail (3 or more criteria met) and pre/non-frail (0-2 criteria met).
^b^ Phenotypes and FRAIL Scale have three levels: frail (3 or more criteria met); pre-frail (1-2 criteria met) or non-frail (0 criteria met).

^c^ Measures of slowness and weakness in the self-reported phenotypes are from self-report questions only (3 questions per criterion)
^d^ Measures in the FRAIL Scale are self-reported for each item, as follows:

- Fatigue: "In the past month, on average, have you been feeling unusually tired during day?" 1 = All of the time, 2 = Most of the time, 3 = Some of the time (or less), 4 = Refused, 5 = Don’t know. Responses of “1” or “2” are scored as 1 and all others as 0.
- Resistance: “For health or physical reasons do you have any difficulty climbing up steps?” 1 = Yes, 0 = No
- Ambulation: "For health or physical reasons, do you have difficulty walking ¼ mile (2-3 blocks)?” 1 = Yes, 0 = No.
- Illnesses: Participants asked about illnesses as part of a medical history questionnaire. The total illnesses (0–13) are recoded as 0–5 = 0 and 6–13 = 1. The illnesses include history of: Arthritis (any); osteoporosis; anemia; cancer; kidney disease; thyroid disease; HBP; high cholesterol; heart disease (any); respiratory disease (any); peripheral vascular disease; diabetes; stroke/tia.
- Loss of Weight: Participants current weight; and report of weight from 1 year ago. Percent weight change is computed as: [[weight 1 year ago - current weight]/weight 1 year ago]] * 100. Percent change ≥ 5% (representing a 5% or greater loss of weight), whether intentional or not, is = 1 and < 5% = 0.
- References:
  - Abellan van Kan G, Rolland YM, Morley JE, Vellas B. Frailty: toward a clinical definition. J Am Med Dir Assoc. 2008;9(2):71-72.
  - Morley JE, Malmstrom TK, Miller DK. A simple frailty questionnaire (FRAIL) predicts outcomes in middle aged African Americans. J Nutr Health Aging. 2012;16(7):601-608.

Bolded text = highest value per column, per category (dichotomized, three-level). Abbreviations: FRAIL=Fatigue, Resistance, Ambulation, Illnesses, Loss of Weight; PFP=Physical frailty phenotype; 95%CI=95% confidence interval; PPV=Positive Predictive Value; NPV=Negative Predictive Value.
